# Urethral advancement and glanuloplasty versus tubularized incised plate urethroplasty for distal hypospadias repair: a prospective randomized study

**DOI:** 10.1186/s12894-023-01242-5

**Published:** 2023-04-28

**Authors:** Basem A. Fathi, Ahmed A. Elgammal, Osama M. Ghoneimy, Ahmed A. Alrefaey, Tamer A. Abouelgreed, Mohamed A. Elhelaly, El-Sayed I. El-Agamy

**Affiliations:** Department of Urology, faculty of medicine, Al-Azhar University, Cairo, Egypt

**Keywords:** Urethral advancement, TIP, Hypospadias, Pediatrics

## Abstract

**Background:**

About one in 200 to one in 300 male births has hypospadias. The prevalence of this congenital anomaly varies worldwide. The meatus is located distally in approximately 70% of cases. Several surgical techniques were described for distal hypospadias repair; despite this, there is no ideal approach. This study compares urethral advancement &glanuloplasty, and TIP techniques in terms of feasibility, duration of operation, and complications. **Patients and**.

**Methods:**

This prospective randomized comparative study was conducted at Al-Azhar University Hospitals from April 2022 to October 2022. Fifty-seven cases with different types of hypospadias were assessed for eligibility. Among them, seven cases were excluded due to the presence of severe chordee (n = 3), proximal variant (n = 2), and recurrent cases of hypospadias (n = 2). Fifty cases were randomly divided into two groups using a 1:1 ratio (computer-generated randomization). Twenty-five cases were subjected to urethral advancement and glanuloplasty, and the rest were subjected to tubularized incised plate (TIP) urethroplasty.

**Results:**

The mean age of all studied cases was 4.2 years. Approximately 52% had coronal or sub-coronal meatus, whereas 48% had glandular meatus. Both groups were matched according to age and meatus location (p > 0.05). No statistically significant difference was observed between the two groups regarding duration of operation, postoperative pain, and postoperative hospital stay. In addition, both groups did not differ significantly in late complications (meatal stenosis, meatal retraction, fistula, and glans dehiscence).

**Conclusions:**

Both urethral advancement &glanuloplasty, and TIP urethroplasty have comparable short-term outcomes. Urethral advancement and glanuloplasty is preferred in certain conditions, especially in circumcised children or those with a narrow urethral plate.

**Trial registration:**

The study protocol was approved by the Pan African Clinical Trials Registry (number for the registry is: PACTR202211757905870) on (29/11/2022). All procedures were performed per the Helsinki Declaration.

## Background

About one in 200 to one in 300 male births has hypospadias. The prevalence of this congenital anomaly varies worldwide; for example, in Europe, 19.9 per 10,000; in North America, 34.2 per 10,000 and in Africa, 5.9 per 10,000. The meatus is located distally in approximately 70% of cases that happened most probably because embryologically, the distal penile urethra ends typically at the sub-coronal area, and then the glans channel from the tip downward fused with the penile urethra [1–3]. Hypospadias is a maldevelopment of the urethral fold and ventral foreskin, with or without penile curvature [4]. There is no single satisfactory way of classifying hypospadias. The most commonly used one depends on the meatus’s preoperative location (glandular or distal penile, mid-penile, and severe proximal types). There were many limitations in this classification, and any modern classification must take into consideration the meatal location (especially after degloving of the penis), foreskin (well/poorly developed/absent), glans and groove configuration (shallow and conical or deep and well developed), urethral plate (well developed or hypoplastic, broad or narrow) penile size (normal or reduced) and curvature (present or absent and the degree)[5]. Diagnosis is generally made after birth; however, prenatal ultrasonography can be used to identify hypospadias individuals with a flattened distal tip of the penis as a characteristic symptom (representing the dorsal hooded foreskin after delivery). A karyotype is required in severe hypospadias, and coexistent testicular maldescent is needed to identify sex development disorders [6]. Beck first introduced urethral advancement for hypospadias repair in 1898. The procedure was not consistently successful because the urethra was not mobilized widely, and many patients had chordee postoperatively. Belman, in 1977 reported on a technique with wide urethral mobilization and advancement for hypospadias repair. Recently, various techniques for urethral advancement have been reported, mostly for distal hypospadias repair where the urethra can either be placed over the corpora cavernosa after incision of the glans ventrally or tunnelled into the glans, and the incidence of complications was less with those modifications than the original one described by Beck with also, limited urethral mobilization and better cosmetic appearance of the glans. All of them aimed to use the native urethra instead of creating new one to decrease the incidence of complications [7, 8]. Another widely accepted technique is tubularized incised plate (TIP) repair, described by Snodgrass in 1994. This method creates a midline incision in the urethral plate. Many centers have reported excellent results using this technique. Because of its ease of use, the TIP approach has gained widespread acceptance. In addition, it has a low incidence of complications and a good cosmetic appearance after the operation [9, 10]. As in all hypospadias surgeries, distal hypospadias repair is not a minor procedure. The position of the meatus is not the only factor determining the difficulty of reconstruction. Distal hypospadias repair can be complex and challenging if there are small-sized glans, narrow and shallow urethral plates, associated curvature, and hypoplasia of the proximal spongiosum. Distal hypospadias repair should be as simple as possible to achieve good functional, cosmetic, and satisfactory outcomes [11]. In this study, we aim to compare urethral advancement &glanuloplasty, and TIP techniques for distal hypospadias repair in terms of feasibility, duration of operation, and complications.

## Patients and methods

**Fig. 1:**
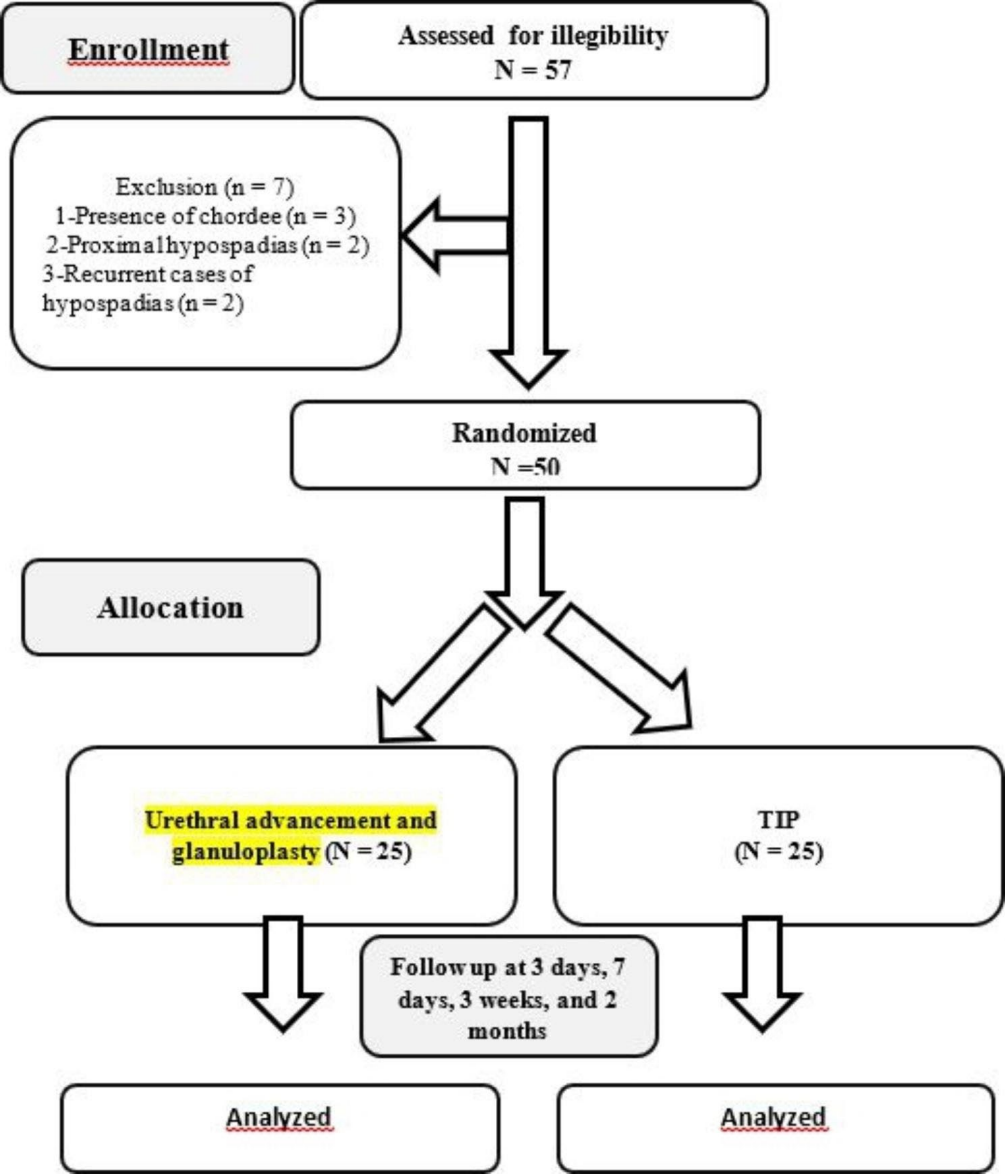
CONSORT flowchart of the study

This prospective randomized comparative study was conducted at Al-Azhar University Hospitals from April 2022 to October 2022 to compare urethral advancement &glanuloplasty and TIP urethroplasty techniques for distal hypospadias repair. Preoperative inclusion criteria included primary cases and absence of severe chordee using eyeball assessment of curvature; intraoperative criteria included the absence of chordee after artificial erection test, while exclusion criteria were recurrent cases, cases with severe chordee, a proximal variant of hypospadias and cases with coagulation disorders. Any case with severe chordee (30 degrees of curvature or more) using eyeball assessment of curvature before the operation or after degloving of the penis and doing an artificial erection test that required any other surgical intervention for correction of chordee was excluded from the study. Only cases with mild chordee resolved entirely by degloving of the penis were included in the study. Fifty-seven cases with different types of hypospadias were assessed for eligibility. Among them, seven cases were excluded due to the presence of chordee (n = 3), proximal variant (n = 2), and recurrent cases of hypospadias (n = 2). Fifty cases were randomly divided into two groups using a 1:1 ratio (computer-generated randomization, single-blind). Twenty-five cases were subjected to urethral advancement and glanuloplasty; the rest were subjected to TIP urethroplasty (Fig. 1). The study protocol was approved by the Research ethics committee of the Faculty of medicine for Girls, Cairo, Al-Azhar University (FMG-IRB), Nasr City, Cairo, Egypt (approval number: 1323) and registered at the Pan African Clinical Trials Registry (number for the registry is: PACTR202211757905870). All procedures were performed under the Helsinki Declaration [12]. Informed consent was obtained from parents prior to the study. All patients’ medical history, physical examination results, and biochemical profiles (including complete urine analysis, coagulation profile, blood urea, and serum creatinine) were obtained. The sample size was calculated using Stata 17 (StataCorp. 2021. Stata Statistical Software: Release 17. College Station, TX: StataCorp LLC.) for the prevalence of hypospadias (1.4–3.3%) reported by Chul Kim et al. [13]. The minimum sample size required was calculated to be 50 using a confidence limit of 5% and power of 80%. Thus, 50 cases were allocated and randomly divided into two groups (25 cases per group).

### Surgical techniques

**Fig. 2:**
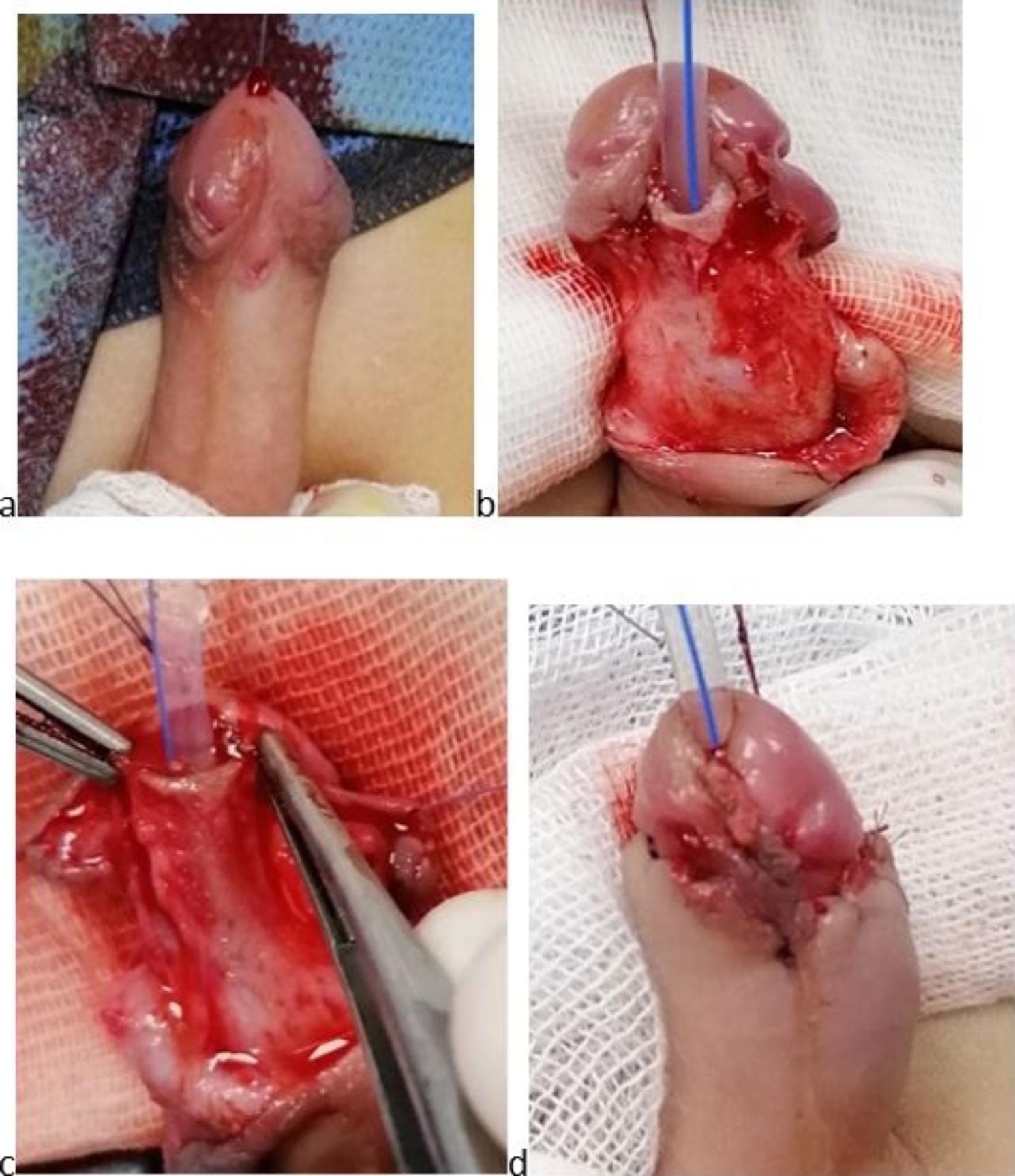
Modified MAGPI: (a) before operation, (b) after degloving, (c) development of glans wings and urethral mobilization, and (d) final appearance after glans and skin closure

**Fig. 3:**
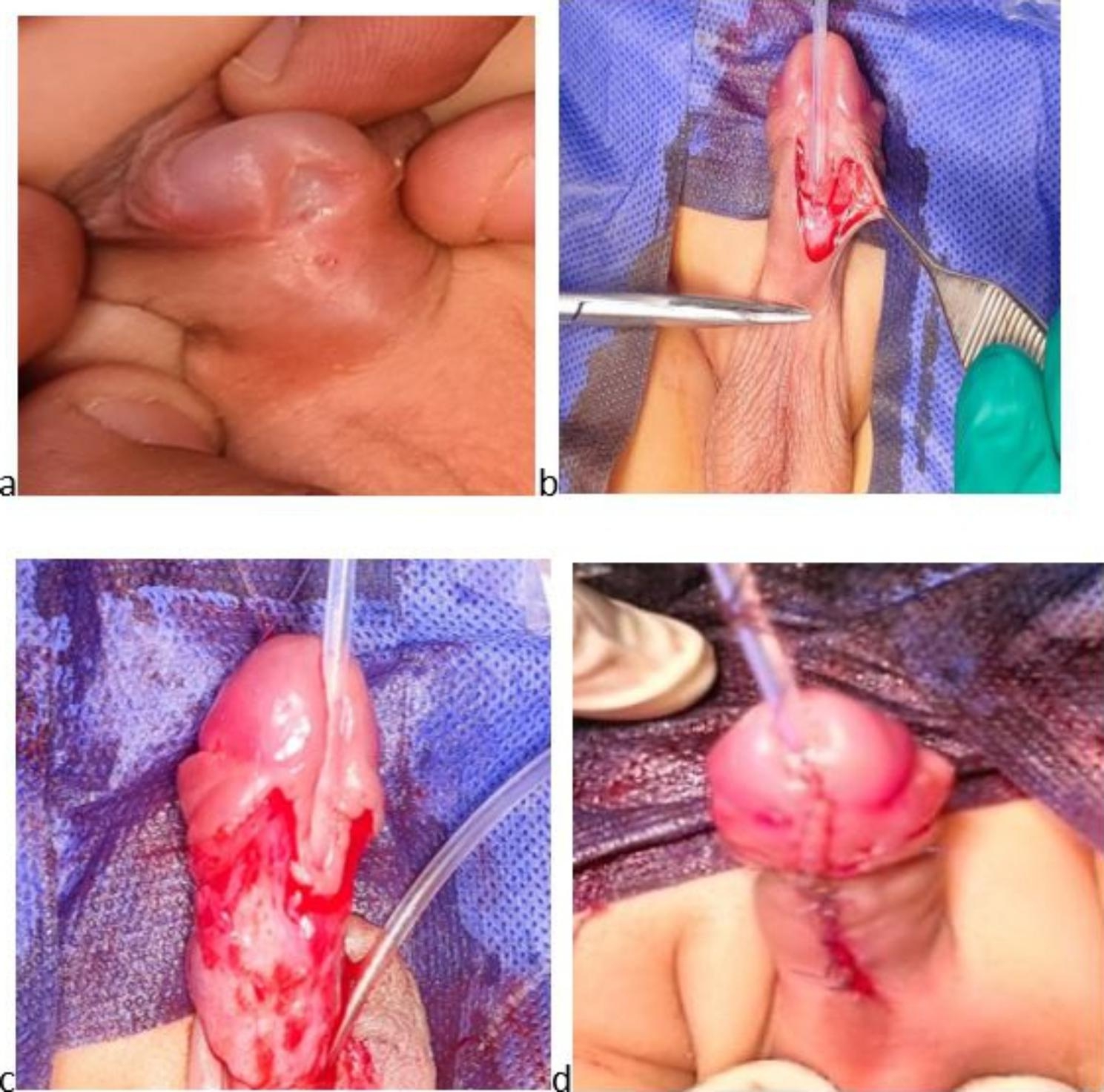
TIP urethroplasty: (a) before operation, (b) U-shaped incision around the urethral plate, (c) after degloving, and (d) final appearance after glans and skin closure

The repair was performed in all patients under general anesthesia with a caudal nerve block in the supine position. First, a traction suture was placed at the glans, and then a silicone urethral catheter was passed (the caliber was selected according to the patient’s age and meatal size). After a circumcising incision, staying under the meatus in the ventral surface of the penis, complete degloving of the penis was done, releasing any tethered tissue causing chordee. Then, after a tourniquet was applied, an artificial erection test was done in all cases to detect the presence of any significant residual chordee. In the urethral advancement and glanuloplasty technique, we performed a meticulous periurethral dissection down to the corpora cavernosa on both sides (about 1–1.5 cm) using a magnifying Loupe (3.5×) and fine instruments. The urethra was then separated from the corpora cavernosa until an appropriate length of the urethra was achieved to reach the tip of the glans without tension. Then, glans wings were developed, and urethral advancement was done. The urethra was fixated in the new site to the glans at 11, 12, 1, 4, and 8 o’clock using a 6/0 polydioxanone suture (PDS) suture. The glans was closed using interrupted mattress sutures with 6/0 Vicryl. Then, the tourniquet was released. After skin closure, the excess foreskin was removed, and circumcision was performed (Fig. 2). A tight dressing was placed for 24 h postoperatively, and a traction suture was used to fix the catheter to the dorsal surface away from the suture line. TIP urethroplasty was performed using the classic technique described by Snodgrass [9]. A 1- to 2-mm incision was made proximal to the meatus, and two parallel longitudinal incisions along the urethral plate were made. Then, glandular wings were developed, and a midline incision of the plate was made down to the underlying corpora from the meatus up to the tip of the glans. Then, tubularization was done using a 6/0 PDS suture around the catheter. A loose vascularized dartos flap from the prepuce and/or the shaft was used as a second layer to cover the neourethra. Then, the glans, skin closure, and dressing were performed as described previously (Fig. 3). The urethral catheter was left 7 days postoperatively in both techniques for fear of glandular edema that happened postoperatively and compressed urethra, causing urinary complaining. except in one case who developed mild wound infection in which the stent was left for three additional days.

### Follow-up

Follow-up was done for all cases in the outpatient clinic at 3 days, 7 days, 3 weeks, and 2 months postoperatively to detect complications. All complications were recorded for analysis. The operators observed the voiding stream force and direction postoperatively to detect any abnormality, and meatal stenosis was confirmed if they failed to pass 6 French catheter through the meatus postoperatively. Success was defined in both groups functionally when the patient had a straight and good urine stream and cosmetically when the patient had glans with a normal conical appearance and orthotopic slit-like meatus.

### Statistical analysis

The collected data were revised, coded, tabulated, and introduced to a personal computer using Statistical Package for Social Science (IBM Corp. Released 2017. IBM SPSS Statistics for Windows, Version 25.0. Armonk, NY: IBM Corp.). Student’s t-test was used to assess the statistical significance of the difference between the two study group means. The Mann–Whitney U test was used to assess the comparison between two nonparametric groups. The chi-square test was used to examine the relationship between two qualitative variables. Statistical significance was considered at p < 0.05 and a 95% confidence interval.

## Results

The mean age of all studied cases was 4.2 years. Approximately 52% had coronal or subcoronal, whereas 48% had glanular meatus (Table 1). Both groups were matched according to age and meatus location (p > 0.05). There was no statistically significant difference between the two groups regarding duration of operation, postoperative pain, and postoperative hospital stay. In addition, late complications (meatal stenosis, meatal retraction, fistula, and glans dehiscence) did not differ significantly between both groups. No significant differences were found between the locations of hypospadias meatus in terms of the studied parameters. Postoperative pain was significantly associated with complications (p = 0.016), whereas age, location, duration of operation, and postoperative hospital stay were not significantly associated with postoperative complications. No cases had postoperative hematoma or infection except in one case in the TIP urethroplasty group who developed mild wound infection in which the stent was left for three additional days and the infection was resolved after treatment with appropriate antibiotics for one week.

**Table 1:** Comparison of studied parameters according to the operation type

			urethral advancement and glanuloplasty technique	TIP urethroplasty	p	Coronal or subcoronal	Glandular	p	No complications	Complications	p
			n = 25 (50%)	n = 25 (50%)		n = 26 (52%)	n = 24 (48%)		n = 36 (72%)	n = 14 (28%)	
**Age (years)**		**mean ± SD**	**4.7 ± 2.7**	**3.8 ± 2.4**	**0.228**	**3.8 ± 1.2**	**4.7 ± 1.2**	**0.240**	**4.2 ± 1.4**	**4.2 ± 1.1**	**0.959**
**Coronal or subcoronal**		**N,%**	**11(44%)**	**15(60%)**	**0.258**	**-**	**-**	**-**	**19(52.8%)**	**7(50%)**	**0.860**
**Glandular**		**N,%**	**14(56%)**	**10(40%)**		**-**	**-**	**-**	**17(47.2%)**	**7(50%)**	
**duration of operation (minutes)**		**mean ± SD**	**55.6 ± 7.5**	**53.8 ± 6.8**	**0.279**	**55.2 ± 7**	**54.2 ± 7.5**	**0.618**	**54.9 ± 7.6**	**54.3 ± 6.2**	**0.802**
**Postoperative pain**		**N,%**	**9(36%)**	**6(24%)**	**0.355**	**6(23.1%)**	**9(37.5%)**	**0.266**	**7(19.4%)**	**8(57.1%)**	**0.016**
**Postoperative hospital stay (days)**		**median, minimum-maximum**	**2(1–3)**	**2(1–3)**	**0.550**	**2(1–3)**	**2(1–3)**	**0.352**	**2(1–3)**	**2(1–3)**	**0.118**
**Postoperative complications**	**Any**	**N,%**	**8(32%)**	**6(24%)**	**0.529**	**7(26.9%)**	**7(29.2%)**	**0.860**	**-**	**-**	**-**
	**meatal stenosis**	**N,%**	**2(25%)**	**3(50%)**	**0.580**	**3(42.9%)**	**2(28.6%)**	**0.577**	**-**	**-**	**-**
	**meatal retraction**	**N,%**	**3(37.5%)**	**1(16.7%)**	**0.580**	**0(0%)**	**4(57.1%)**	**0.070**	**-**	**-**	**-**
	**fistula**	**N,%**	**2(25%)**	**2(33.3%)**	**0.733**	**3(42.9%)**	**1(14.3%)**	**0.559**	**-**	**-**	**-**
	**Glans dehiscence**	**N,%**	**1(12.5%)**	**0(0%)**	**0.369**	**1(14.3%)**	**0(0%)**	**0.299s**	**-**	**-**	**-**

## Discussion

During the research, we found some studies that compared either both techniques or described cohorts for each technique everyone alone, then reported the complications and success rate. Urethral mobilization is a relatively safe procedure as the urethra has good vasculature from the urethral branch of the internal pudendal artery and the dorsal penile artery, with fine communications between them. In addition, the urethra has good elasticity and continuous growth during childhood. Thus, urethral advancement and glanuloplasty can theoretically be performed without fear of affecting the vascularity of the urethra [14]. In their study about the trends in hypospadias surgery, Springer et al. concluded that TIP urethroplasty is preferred in repairing distal variations [10]. This is because the TIP technique is a simple procedure and has a high success rate. The same conclusion was reported by Cook et al. in distal and midshaft hypospadias [15]. In this study, we had three cases of meatal retraction, two cases of meatal stenosis, two cases of urethrocutaneous fistulae, and one case of glans dehiscence in the urethral advancement and glanuloplasty group. In the TIP urethroplasty group, we had one case of meatal retraction, three cases of meatal stenosis, and two cases of urethral cutaneous fistulae. There were eight cases of complications in the urethral advancement and glanuloplasty group (32%) versus six cases in the TIP urethroplasty group (24%). However, the difference was not significant (p = 0.529). Patients with meatal stenosis only required gentle dilatation weekly for four successive weeks. In patients with meatal retraction, the meatal location remained within the limit of the glans and was sufficient to achieve micturition in a straight stream while standing, and no second intervention was needed as there was no complaint of the cosmetic appearance from the parents of those children. Only patients with fistulae (n = 4) and glans dehiscence (n = 1) required a second intervention after 6 months of the primary repair. The two cases that developed fistula in the urethral advancement and glanuloplasty group were most probably caused by injury to the urethra during dissection that was not noticed during the operation. There were no cases of postoperative hematoma and infection (except one case with mild infection). This may be because good hemostasis was done before skin closure, and we frequently use sterile dressing until healing occurs. Many studies discussed their results using the urethral advancement and glanuloplasty technique (with different modifications). All of them were searching for a simple and successful operation with fewer complications as they used the native urethra without creating a new urethra. Macedo Jr et al. treated 164 patients with distal hypospadias using their GUD (glandular urethral disassembly) technique. They found complications in 6 patients (3.6%) consisting of five fistulas (3%) and three glans dehiscence (1.8%) (two of them had both complications). They believed they could not achieve good results only by advancing the urethra without reconstructing the glans, even by aggressive mobilization, as reported by Koff et al. The glans represents a significant component of the hypospadias abnormality, and by repairing it in association with mobilization of the urethra, a much better result can be obtained. They believed that their GUD technique is a viable alternative to distal hypospadias repair as they can perform it with any urethral plate quality as it does not require a minimum glans width as the TIP repair [8, 16]. Harrison and Grobbelaar performed a study on 47 patients. They had one case of fistula that required a second intervention and three cases with meatal retraction. They concluded that the urethral advancement and glanuloplasty technique is safe, leads to an excellent cosmetic outcome, has a low complication rate, and can be performed in circumcised children as the prepuce is not utilized in the procedure [17] Elemen and Tugay reported that the limited urethral mobilization technique for distal hypospadias repair is effective, simple, and results in an excellent functional and cosmetic outcome. In their study, five out of 47 patients developed meatal stenosis. Among them, four patients only required gentle dilation, whereas one needed meatotomy. Another patient had a minor glans detachment [14]. Sixty individuals with distal hypospadias were studied by Haider et al., who then repaired the condition by mobilizing and advancing their urethras. The meatal retraction occurred in two patients (5%), necessitating further treatment in both instances. Meatal stenosis was the cause in four cases (6.6%), and dilatation was all that was required. Five cases had hematoma, and one had an infection; all were managed conservatively. The authors concluded that urethral mobilization and advancement result in good cosmetic and functional outcomes and can be utilized for distal hypospadias repair with a low incidence of complications, especially urethrocutaneous fistula [18]. Edan reported that the urethral mobilization technique is a good choice for distal hypospadias repair. His study was performed on 65 children and excluded those with hypoplastic urethra and proximal variants. Three cases had meatal stenosis (4.6%) and were successfully treated with dilatation. One case developed meatal retraction (1.5%) but did not require a second intervention as the meatus was still within the glans [19]. Our study did not find a significant difference between the two groups concerning the duration of operation, postoperative hospital stay, and postoperative complications (meatal stenosis, retraction, fistula formation, or glans dehiscence). We think that meticulous periurethral dissection in the urethral advancement technique requires nearly the same time needed for neourethral creation and second-layer coverage in the TIP technique, as we noticed during our practice. However, complications were higher in the urethral advancement and glanuloplasty group than in the TIP urethroplasty group. In certain conditions, especially in circumcised children and those with shallow or narrow urethral plates, urethral advancement and glanuloplasty may be an alternative solution.

## Limitations

Despite being a prospective study, the present study has some limitations. First, there were two surgeons and not only one who performed the operations in both groups. The surgical skills may differ from one to another and may result in less favorable outcomes. Second, the follow-up time was short. In some cases, the follow-up time was 2 months as the parents thought there was no longer a need for more follow-up given that no apparent complications appeared. Thus, further studies with long-term follow-up are needed to detect any late complications. Third, our study was performed in a single center. A multicenter study will be planned to obtain more information and provide more accurate results.

## Conclusions

Our study showed that urethral advancement &glanuloplasty, and TIP urethroplasty have comparable short-term outcomes. Urethral advancement and glanuloplasty is preferred in certain conditions, especially in circumcised children or those with a narrow urethral plate. Further studies are needed to confirm our findings and identify the preferable technique in those cases.

## Data Availability

The dataset of the current study is available from the corresponding author upon reasonable request.

## List of Abbreviations

TIP: Tubularized incised plate
PDS: Polydioxanone suture
GUD: Glandular urethral disassembly
